# Over-indebtedness as a marker of socioeconomic status and its association with obesity: a cross-sectional study

**DOI:** 10.1186/1471-2458-9-286

**Published:** 2009-08-07

**Authors:** Eva Münster, Heiko Rüger, Elke Ochsmann, Stephan Letzel, André M Toschke

**Affiliations:** 1 Institute of Occupational, Social, and Environmental Medicine, University of Mainz, Mainz, Germany; 2 Institute of Occupational and Social Medicine, University of Aachen, Aachen, Germany; 3 King's College London, Division of Health and Social Care Research, Department of Public Health Sciences, London SE1 3QD, UK

## Abstract

**Background:**

The recent credit crunch will have implications for private households. Low socioeconomic status is associated to various diseases. While income, education and occupational status is frequently used in definitions of socioeconomic status, over-indebtedness of private households is usually not considered. Over-indebtedness is currently increasing in high-income countries. However, its association with health – particularly with obesity – remains unknown. Therefore, the aim of this study was to assess an association between over-indebtedness and overweight or obesity.

**Methods:**

A cross-sectional study on over-indebtedness and health including 949 over-indebted subjects from 2006 and 2007 in Rhineland-Palatinate and Mecklenburg-Western Pomerania (Germany) and the telephonic health survey 2003 of the Robert Koch-Institute including 8318 subjects, who are representative for the German population, were analysed with adjusted logistic regression considering overweight (BMI ≥25.0 kg/m^2^) and obesity (BMI ≥30 kg/m^2^) as response variable.

**Results:**

After adjusting for socio-economic (age, sex, education, income) and health factors (depression, smoking habits) an independent effect of the over-indebt situation on the probability of overweight (aOR 1.97 95%-CI 1.65–2.35) and obesity (aOR 2.56 95%-CI 2.07–3.16) could be identified.

**Conclusion:**

Over-indebtedness was associated with an increased prevalence of overweight and obesity that was not explained by traditional definitions of socioeconomic status. Over-indebtedness should be additionally considered when assessing health effects of socioeconomic status.

## Background

The recent credit crunch will affect public and private health services in various ways and there is already evidence of current cutbacks [1]. The turmoil in the banking system may also affect charitable healthcare providers. Naomi House children's hospice in Winchester (UK), for example, faces the potential loss of nearly $10 m it has invested in the troubled Icelandic bank Kaupthing Singer and Friedlander [2]. Other than that mentioned the financial crises will influence individuals' every day life with consequences for the individuals' health.

A remarkable increase in the number of over-indebted people in European countries and the US can currently be observed [3]. For example, in Germany, about 3 million private households (7.6%) corresponding to more than 6 million residents are currently over-indebted [4,5]. Over-indebtedness can be defined as lack of possible debt redemption in due time due to the relation of income and cost of living after a remarkable cutback in standard of living [6].

The link between socioeconomic status and health is well documented, particularly for overweight [7-12]. However, current definitions of socioeconomic status do not consider over-indebtedness and the effect of over-indebtedness on health remains unknown.

Therefore we examined a possible association between over-indebtedness and overweight in adults in Germany.

## Methods

Data on over-indebted individuals (n = 949) from a cross sectional study were considered together with the German National Telephonic Health Interview Survey of the Robert Koch-Institute (n = 8318), which is representative for the German population.

### a) German National Telephone Health Interview Survey 2003 conducted by the Robert Koch-Institute (GNT-HIS)

Details on the survey have recently been published [13]. In brief, the GNT-HIS 2003 is a nationally representative health survey of the adult population in Germany with computer assisted telephone interviews (n = 8318) covering various aspects of diseases, including risk factors, quality of life, health care utilisation and socioeconomic status. The response rate of the GNT-HIS 2003 was 52.3%.

Ethics review board approval was not obtained for these secondary analyses of an existing public access dataset.

### b) A cross-sectional study regarding over-indebted individuals (OI-survey)

This study was performed in order to measure the health status of over-indebted individuals and their participation in all aspects of society and their utilization of the health care system. It is a survey on over-indebted residents of the German states Rhineland-Palatinate and Mecklenburg-Western Pomerania. An anonymous survey was organised among clients of debt counselling centres. The survey was carried out by the centres for debt and insolvency counselling of Rhineland-Palatinate and Mecklenburg-Western Pomerania and by the centre for debt counselling of the Johannes Gutenberg-University Mainz in 2006 and 2007. Overall, 949 over-indebted subjects were interviewed (participation rate 39.7%). In the OI-survey we did not use reminder-actions in non-responders. Details on the survey have recently been published [14]. Ethical committee approval was obtained. Informed consent was waived to assure anonymity.

Socio-demographic parameters (age, sex, income, education), Body-Mass-Index, smoking behaviour and depression were obtained in both surveys and used for the analyses. All participants of the telephonic health survey were categorised as not over-indebted, while all OI-survey-participants were categorised as over-indebted. Although a bias towards the null cannot be ruled out, this procedure was chosen due to lacking information on debts in the telephonic health survey.

Participants with missing data of Body-Mass-Index (*GNT-HIS *n = 179; OI-survey n = 8) were excluded for analyses. The joint database (GNT-HIS and OI-survey) contained 9080 data records (941 data records of the OI-survey and 8139 data records of GNT-HIS).

Participants' self reports on height and weight were used to calculate the Body-Mass-Index in order to define the target value "overweight" and „obesity“. The WHO classifications for overweight (≥25.0 kg/m^2^) and obesity (≥30.0 kg/m^2^) were used [15]. Data on participants without overweight or obesity were used as reference.

The prevalence of overweight and obesity associated with over-indebtedness was calculated.

As potential confounders from the literature sex, age, education, income, depression and smoking habits were considered. In crude analysis, they were a priori coded by using binary dummy variables, to improve understanding.

Proportions and their 95%-confidence intervals of dependent variables and potential confounders were calculated. Corresponding unadjusted odds ratios and their 95 percent confidence limits for the association between overweight or obesity and the potential confounders were calculated by using logistic regression analysis.

In multiple logistic regression analysis, all potential confounding factors were modelled in their original categorical form or by using the original binary dummy variables (sex, smoking). They were hierarchically entered to assess their cumulative influence on the association between the risk factor "over-indebtedness" and overweight/obesity.

All calculations were carried out with the software package SPSS (SPSS Inc., Chicago, Illinois), version 14.0

## Results

### Characteristics of study participants

An association between over-indebtedness and gender was not observed (table 1). On average over-indebted participants were younger, with lower education and income, and had a higher prevalence of depression, overweight, obesity and daily tobacco consumption compared to the general population (table 1).

**Table 1:** Prevalence of overweight, obesity and potential confounders within general and over-indebted population

	GNT-HIS (general population) n = 8318		OI-survey (over-indebted population) n = 949	
	
	% with parameter present	95% CI	% with parameter present	95% CI
Overweight	44.3	43.2–45.3	56.8	53.6–59.9
Obesity	11.3	10.6–12.0	25.1	22.3–27.8
				
Sex (male)	46.5	45.5–47.6	47.0	43.8–50.2
Age (>40 years)	62.1	61.1–63.1	52.9	49.7–56.1
Education (≥10 years)	68.3	67.3–69.3	40.0	36.9–43.2
Income, net (>1500 €)*	54.4*	53.3–55.5	22.6	19.9–25.2
Depression (yes)	9.4	8.8–10.0	38.9	35.8–42.0
Smoking (yes)	33.9	32.9–34.9	63.0	55.9–66.1

Notes: in crude analysis, covariates were dichotomised to improve understanding

*2134 subjects gave no information about income. By excluding them from the dataset the appropriate portion value for this character would be 73.2%

### Potential confounders

Unadjusted odds ratios for the association between possible confounders and overweight/obesity are shown in table 2. A higher risk of being overweight or obese was observed for male sex, and age above 40 years as well as for being depressive. Subjects reporting a higher education, higher income, and smokers had a lower risk of overweight or obesity (table 2).

**Table 2:** Unadjusted odds ratios for the association between possible confounders and overweight/obesity

	Overweight		Obesity	
	Odds ratio	95% CI	Odds ratio	95% CI

Sex (male)	1.96	1.80–2.13	1.15	1.02–1.30
Age (>40 years)	2.55	2.34–2.79	2.00	1.74–2.30
Education (≥10 years)	0.47	0.43–0.51	0.46	0.41–0.52
Income, net (>1500 €)	0.88	0.80–0.97	0.67	0.58–0.76
Depression (yes)	0.98	0.87–1.11	1.50	1.27–1.77
Smoking (yes)	0.68	0.63–0.74	0.74	0.66–0.85

Notes: in crude analysis, covariates were dichotomised to improve understanding; n = 9080

### Confounder adjusted estimates

After adjustment the association between over-indebtedness and body composition had an odds ratio of 1.97 (95%-CI 1.65 to 2.35) for overweight and 2.56 (95%-CI 2.07 to 3.16) for obesity (table 3). Adjustment for socioeconomic status variables such as education or income did not explain this association (table 3).

**Table 3:** Unadjusted and adjusted odds ratios for the association between over-indebtedness and overweight/obesity*

	Overweight		Obesity	
	Odds ratio	95% CI	Odds ratio	95% CI

Over-indebtedness (unadjusted)	1.60	1.40–1.83	2.54	2.16–2.99
Adjusted for				
Sex and age	1.98	1.71–2.29	2.91	2.46–3.44
+ Educational level	1.65	1.42–1.93	2.42	2.03–2.90
+ Income level	1.76	1.49–2.09	2.41	1.97–2.95
+ Depression	1.81	1.52–2.15	2.34	1.90–2.87
+ Smoking	1.97	1.65–2.35	2.56	2.07–3.16

Notes: *confounders and independent risk factors modelled in ordinal, polytomous categorical form; n = 9080

## Discussion

A higher risk of obesity was observed for over-indebted individuals compared to the general population. This association was not explained by components of traditional socioeconomic status definitions such as education and income as well as by other characteristics including sex, age, depression, and smoking habits. This explorative finding suggests an independent association between over-indebtedness and overweight or obesity.

These results are unlikely due to differences in the composition of the study collectives of both surveys. Rather more these findings are in accordance with international models assuming a link between individual's financial situation and the diversity of access to „healthy“ food [16-18]. An inverse relationship between energy density of food and its costs combined with the inability to pay can be a partial reason for the higher risk of overweight or obesity among over-indebted subjects.

Apart from the shortness of financial resources and possible associations with obesity, over-indebted individual's psychological distress has to be considered. A depressed emotional state can lead to increased food intake (hyperphagous reaction hypothesis) [19]. In the situation of over-indebtedness, eating can become a compensation and gratification. The subsequent positive feeling might be a substitute for other deficits.

The remarkably increased risk of obesity among the group of occasional and former smokers as well as among non-smokers compared to daily smokers might also be a sign for surrogate behaviour. Smoking has been shown to affect Leptin levels that are associated with satiety and food intake as well as smoking increases the basic metabolic rate possibly explaining these findings [20-22].

It might belong to a poor stress management based by the financial strain of over-indebtedness to lapse stronger into non-health-conscious behaviour like „smoking“ or „increased food intake leading to obesity“ [23].

The choice of food is mainly determined by personal taste, cost and convenience and less by health aspects or the will to maintain a well-balanced diet [24]. Increasing the availability of „healthy“ food by low-pricing campaigns could be an effective public health measure.

Over-indebtedness affects a series of risk factors for chronic diseases such as leisure time activities as well as participation in social activities [25]. Similarly, the diet might be limited in quality. The quality of an individual's diet often depends on financial resources and the ability to choose food [26-28] possibly boosting the overweight pandemic in low socio-economic groups [16,29]. Energy-dense food such as sweets or fatty snacks are often less expensive [30] compared to food with lower energy density such as fruit or vegetables [18,31].

The financial situation of over-indebted private households can partly account for the high prevalence of obesity among this group of persons. Lower quality of life deriving from deprived economic, social and environmental circumstances as well as behavioural risk factors and limited access to participation in daily life have to be taken into account when considering the complexity and diversity of the causes of obesity.

A couple of methodological issues have to be considered. First, overweight or obese people might have difficulties in finding a job or might have lower salaries than normal-weight individuals and thus may be more prone to over-indebtedness [32,33]. Such a reverse causation cannot be ruled out [34]. Second, in the general population sample, some subjects might have been over-indebted, possibly underestimating the true association between over-indebtedness and obesity reported in this study. Third, self-reported weight and height were used for these for analyses [35-37]. Self-reporting of weight and height might result in reporting bias. A potential non-differential misclassification cannot be ruled out and might have attenuated the association between over-indebtedness and overweight or obesity. A differential misclassification might result in different prevalences of overweight or obesity. However, this is similar to a change of the cut-off values for overweight or obesity and it has been shown that a change in cut-off values still allows assessment of relationships [38]. Fourth, one of the states (Mecklenburg-Western Pomerania) included in the survey on over-indebted people has one of the highest rates of unemployment in Germany. Non-indebted subjects from this state may have a lower income and higher prevalence of obesity when compared with Germany in total. However, since adjustment for income did not explain the association between over-indebtedness and obesity, confounding due to income seems to be unlikely.

## Conclusion

The results of the present study illustrate that apart from traditional socioeconomic factors over-indebtedness is associated to health in terms of body composition. Definitions of socioeconomic status used for health research should also consider the dept situation.

## Competing interests

The authors declare that they have no competing interests.

## Authors' contributions

EM, SL conceptualised the study and developed the study protocol of the OI-survey. EO, HR and AMT were responsible for the analysis. EM and AMT wrote the initial draft of the paper, which was subsequently modified in discussions with all authors. EM is the guarantor of the work. All authors read and approved the final manuscript.

## Pre-publication history

The pre-publication history for this paper can be accessed here:

http://www.biomedcentral.com/1471-2458/9/286/prepub
